# Challenges and Mitigation Strategies in the Development and Feasibility Assessment of a Digital Mental Health Intervention for Depression (VMood): Mixed Methods Feasibility Study

**DOI:** 10.2196/68297

**Published:** 2025-06-30

**Authors:** Leena W Chau, Jill K Murphy, Vu Cong Nguyen, Hai Nhu Tran, Harry Minas, Raymond W Lam, Kanna Hayashi, Thi Thanh Xuan Nguyen, Emanuel Krebs, John O'Neil

**Affiliations:** 1 Simon Fraser University Burnaby, BC Canada; 2 St. Francis Xavier University Antigonish, NS Canada; 3 Institute of Population, Health and Development Hanoi Vietnam; 4 University of Melbourne Melbourne Australia; 5 University of British Columbia Vancouver, BC Canada; 6 BC Cancer Agency Vancouver, BC Canada

**Keywords:** digital mental health, depression, implementation science, usability, low- and middle-income countries, LMICs, Vietnam

## Abstract

**Background:**

Worldwide, the COVID-19 pandemic contributed to further gaps in mental health care, particularly in low- and middle-income countries such as Vietnam, where care is inaccessible for 90% of those who need it. There has subsequently been a considerable increase in the use of digital mental health interventions such as smartphone apps. Presently, the evidence for such interventions is limited, especially in cases in which the interventions have been adapted from evidence-based in-person formats. Implementation science aims to promote the incorporation of scientific findings into practice. A key determinant of implementation success is an intervention’s usability. Hurdles to usability include an intervention being too confusing or time-intensive to use. Facilitators include incorporating a greater number of engagement features and integrating human support.

**Objective:**

The aim of this implementation science feasibility study was to describe the challenges and mitigation strategies used in the development, usability testing, and implementation of a digital depression intervention (VMood smartphone app) developed in Vietnam. VMood was adapted from an evidence-based in-person intervention originally developed in Canada that is grounded in principles of cognitive behavioral therapy with supportive coaching by a lay health or social services worker. The research team is currently testing the effectiveness and cost-effectiveness of VMood in a randomized controlled trial across 8 provinces in Vietnam informed by the results of this feasibility assessment.

**Methods:**

This mixed methods feasibility study was organized using an implementation outcome framework focused on acceptability, adoption, appropriateness, and feasibility. This study involved three data collection components: (1) usability testing (interviews and focus groups with app user and provider participants who tested VMood in 1 Vietnamese province), (2) app metrics (from the early phase of the randomized controlled trial in the same province but from different municipalities), and (3) discourse data (notes from various team meetings, communications, and reports on VMood’s development and implementation). Qualitative data were analyzed using thematic content analysis. App use data were analyzed using basic descriptive statistics.

**Results:**

The findings of the 3 data components showed that there were seven main challenges: (1) challenges with recruitment and uptake of the app, (2) challenges with use and engagement, (3) screening challenges, (4) digital divide, (5) limitations to digital applications for mental health, (6) technological challenges, and (7) funding and policy constraints. Various solutions to help mitigate the challenges were used by the team.

**Conclusions:**

The findings contribute important evidence on the challenges to the development and feasibility assessment of a digital depression app adapted from an in-person intervention in Vietnam. The findings have applicability for others looking to develop and implement digital interventions in similar contexts, serving as a unique opportunity to share the lessons learned regarding the development and testing process.

## Introduction

### Background

Mental disorders are a leading cause of global disease burden [1], with approximately 1 in every 8 people living with a mental disorder in 2019 [2]. Improving access to mental health care as a global priority was further magnified in the context of the COVID-19 pandemic, which has contributed to deteriorations in population mental health [3]. The Global Burden of Disease 2020 study estimated that the pandemic has led to increases of 28% in major depressive disorders and 26% in anxiety disorders [3], 2 of the most common mental disorders [4]. The COVID-19 pandemic has also led to severe disruptions to already limited in-person services [5]. This has had a profound impact worldwide, including in low- and middle-income countries such as Vietnam [6-8]. Recent evidence shows the prevalence of mental disorders in Vietnam to be of 10.1 million (rate of 10.2%), with the prevalence (and rates) of depression and anxiety being 2.7 million (3%) and 2.9 million (2.7%) [9], respectively, based on the population of 98.9 million in 2021 [10]. This is especially concerning in Vietnam as mental health care is inaccessible for up to 90% of those who need it [11]. In response, there has been a considerable increase in mental health interventions delivered through digital means [12] such as smartphone apps [13] as they have emerged as a promising way to expand access to care within the additional constraints imposed by the COVID-19 pandemic. Presently, the evidence for such interventions is limited [14,15].

Implementation science aims to promote the incorporation of scientific findings and other evidence-based approaches into practice to improve health services [16]. A key determinant of implementation success is an intervention’s usability [17]. Usability is the extent to which a product can be used easily, effectively, and with satisfaction to obtain specified goals [18]. Usability has been commonly referred to as a key factor in the successful implementation and sustainability of digital health apps [19], with poor usability being a primary reason for failed adoption [20]. Approximately one-third of users who download health apps stop using them after a short time [21]. An additional consideration for individuals with depression is specific symptoms such as low motivation, concentration difficulties, and behavioral avoidance that may make it more difficult for them to engage with digital health interventions [22], posing further challenges to real-world effectiveness. Facilitators of usability include incorporating a greater number of engagement features (eg, praises and reminders) [23] and integrating human support [24].

### Prior Work

VMood has been adapted from an in-person supported self-management (SSM) intervention originally developed in Canada [25]. This intervention is grounded in principles of cognitive behavioral therapy (CBT) and uses an SSM approach consisting of an *Antidepressant Skills Workbook* (ASW) and supportive coaching delivered in person by a lay health or social services worker (eg, community worker or peer navigator). SSM has been shown to be effective in the Vietnamese context through a previous randomized controlled trial (RCT; funded by Grand Challenges Canada; 2016-2019) conducted by this research team across 8 Vietnamese provinces [26]. In response to the Government of Vietnam’s budgetary changes and the restrictions on in-person care imposed by the COVID-19 pandemic, the team accepted the request from the Vietnamese Ministry of Labour, Invalids, and Social Affairs (MOLISA) to adapt the intervention to a digital format rather than scale up the in-person SSM intervention as originally planned. This work is funded by the Canadian Institutes of Health Research and Grand Challenges Canada (with matching funding provided by MOLISA).

### Ongoing Work

VMood, delivered via a smartphone app, comprises 3 months of engagement with the VMood program, which includes the various modules (eg, *Introducing depression*, *Antidepressant skills*, and *Reactivating your life*) from the ASW being delivered in an interactive multiple-choice format. Each module includes an introductory video with a text option. The VMood program also includes support delivered through the app via messages from a social worker. Social workers, a relatively new profession in Vietnam that received official designation as a profession in 2010 [27], work under the purview of MOLISA [27]. The profession is expanding in Vietnam, reflected in the number of schools providing social worker training increasing from 2 in 2003 to 35 in 2024 [28]. Social workers were envisioned to play a role in the delivery of mental health care in community-based settings but have in general received minimal mental health training [29]. Participating social workers will be trained on basic depression, addressing stigma, and providing coaching via the app through a web-based training program developed by the Institute of Population, Health, and Development (PHAD). PHAD is a leading nongovernmental research institute in Vietnam and the research team’s implementing partner.

In Vietnam, responsibility for mental health is shared between MOLISA and the Ministry of Health. MOLISA oversees 24 long-term care centers for individuals with severe mental health conditions attached to their provincial Social Protection Centers (SPCs), whereas the Ministry of Health oversees 36 psychiatric hospitals and 25 psychiatry departments within provincial general hospitals [30]. Despite a policy shift since 1998 toward a community-based approach to mental health care with the focus on prevention and delivering low-barrier care to those with common mental disorders such as mild to moderate depression [29,31,32], progress has been slow. Vietnam’s mental health system still comprises mainly large tertiary hospitals providing care only for those with severe mental health conditions using predominantly pharmacological treatment, with very little to no community-based care available for those with mild to moderate depression [33].

In this context, there is an added urgency to expand community-based mental health care for the large and growing population of those who need it. There is, at present, limited critical evidence on the unique considerations and practical guidance for the development and implementation of digital apps for depression adapted from evidence-based in-person formats. This paper will describe the challenges encountered in the development and feasibility assessment of VMood in real-world settings in 1 province of Vietnam and mitigation strategies that the team used to address them. At the time of writing, the larger RCT is being implemented across the 8 participating provinces in Vietnam informed by the lessons learned described in this paper. The findings have applicability for others looking to develop and implement digital interventions in similar contexts.

### Goal of This Study

The aim of this implementation science feasibility study was to describe the challenges in the development and feasibility assessment of a digital depression intervention (VMood smartphone app) adapted from an in-person format in Vietnam, along with mitigation strategies regarding how the research team addressed them. Specific objectives were as follows:

1. To describe the challenges in the process of adapting an in-person depression intervention to a digital format (VMood app) and the challenges encountered in VMood’s feasibility assessment
2. To describe the strategies that the team used to mitigate and address the challenges and the lessons learned

## Methods

### Overview

This feasibility assessment used mixed methods to examine the challenges influencing the development, usability testing, and implementation of the VMood digital depression intervention in Vietnam and describe the strategies used by the research team to mitigate these challenges. The team comprises researchers from diverse backgrounds (eg, global mental health, anthropology, psychiatry, statistics, and computer science) in Vietnam, Canada, and Australia. In total, 3 data components informed the findings (Table 1). The overall time frame for the feasibility study was from the beginning of app development (January 2021) to the end of the first phase of the RCT in 1 province in Vietnam (June 2024).

**Table 1:** Data collection components for the overall feasibility study conducted from June 2023 to March 2024.

Data type	Description	Sources
Usability testing	Early testing of the app to gather data on usability and acceptability. Conducted in Thanh Hoa province from June 2023 to September 2023	Interviews and focus groups
App metrics	Data on rates of enrollment, log-ins, and frequency and duration of engagement with specific components of the app to supplement qualitative usability testing results. Gathered from Thanh Hoa from December 2023 to June 2024	Use data from the VMood app Due to technological challenges with VMood, app metrics data were not available from the usability testing and were instead gathered from the first phase of the RCT^a^, also conducted in Thanh Hoa; the RCT involves different regions (ie, the participants are different from those in the usability testing)
Discourse data	Notes from various meetings and emails that describe the challenges in the development, usability testing, and implementation of VMood, along with reports from the early phase of RCT implementation in Thanh Hoa	Meeting notes (monthly team, small-group, and internal meetings) Personal communications (emails) Field trip report from first phase of RCT implementation in Thanh Hoa (January 2024) Progress reports from the Thanh Hoa RCT (January 2024-March 2024)

^a^RCT: randomized controlled trial.

### Conceptual Framework

The feasibility study was informed by the implementation outcomes framework by Proctor et al [34], focusing on *acceptability*, *adoption*, *appropriateness*, and *feasibility* (Textbox 1). Additional outcomes—*costs*, *penetration*, and *sustainability*—are beyond the scope of this study, and the last outcome, *fidelity*, is captured in more detail elsewhere [35]. This framework evaluates implementation outcomes, which are distinct from clinical outcomes examining an intervention’s effectiveness. However, they serve as intermediate outcomes to clinical outcomes because, if an intervention is not implemented well, it will likely not be clinically effective [34]. Implementation science frameworks are different from theories, sets of analytical statements about relationships between constructs, and models, a simplification of a specific aspect of a more complex world in the sense that frameworks provide a series of steps summarizing how the implementation process should be planned, carried out, and evaluated [36]. The framework by Proctor et al [37] was chosen because it enabled us to selectively focus on outcomes that are important in the early stages of the implementation process.

**Conceptual framework—selected implementation outcomes from the implementation outcomes framework by Proctor et al. Definitions are taken directly from the work by Proctor et al.**

**Implementation outcome and definition**

- Acceptability: “Perception among implementation stakeholders that a given treatment, service, practice, or innovation is agreeable, palatable, or satisfactory”
- Adoption: “Intention, initial decision, or action to try or employ an innovation or evidence-based practice”
- Appropriateness: “Perceived fit, relevance, or compatibility of the innovation or evidence-based practice for a given practice setting”
- Feasibility: “Extent to which a new treatment, or an innovation, can be successfully used or carried out within a given agency or setting”

### Procedures

#### Usability Testing

The usability testing component took place across 6 communes (municipal subdivisions) in 1 district of the Thanh Hoa province in Vietnam. The names of the communes and district have been omitted for confidentiality purposes. The communes were selected in collaboration with MOLISA to represent diversity in terms of economic status (eg, urban and rural) and population composition (eg, older and younger). Usability testing involved participants testing the VMood app followed by the collection of qualitative data (interviews and focus groups). Project activities were conducted at the Thanh Hoa provincial SPC or in the community. Table 2 provides a summary of the participant categories.

**Table 2:** Overview of participants of the usability testing conducted between June 2023 and September 2023.

Category and description			Source (participant code)
**Policy stakeholder (n=1)**			
	The director of the provincial SPC^a^. Responsible for providing oversight in project implementation in the province.	Interview (IPS-01)	
**Providers (n=15)**			
	2 social workers based in the SPC and responsible for project administration: 1 project coordinator responsible for all project activities and 1 provincial coordinator who served as the focal point for requests and recommendations from the province to the project team	Interviews (IPR-01 and 02)	
	13 community women’s union staff members (ie, lay workers) based in the communes who are primarily volunteers active in their communities supporting social and health initiatives. Responsible for helping app users navigate the app.	Focus groups (FGPR-01 [n=8] and FGPR-02 [n=5])	
**App users (n=6)**			
	With depression caseness as defined by a PHQ-9^b^ [38] score of ≥5	Interviews (IAU-01 to 06)	
**Clinical experts (n=5)**			
	Familiar with CBT^c^ principles and depression—2 from Vietnam and 3 from Canada	Interviews (IEX-01 to 05)	

^a^SPC: Social Protection Center.

^b^PHQ-9: 9-item Patient Health Questionnaire.

^c^CBT: cognitive behavioral therapy.

Our research team worked with MOLISA to have an individual appointed as the main national MOLISA coordinator who would facilitate recruitment for the overall study. The policy stakeholder, along with the 2 social workers based at the Thanh Hoa SPC, were recruited through official government channels by the MOLISA coordinator. The community women’s union staff members (lay workers active in their communities; n=13) based in the communes were recruited by the Thanh Hoa project coordinator to support app deployment. PHAD team members administered the training program to the intervention providers in person. App users who self-identified as experiencing symptoms of depression were recruited from the community through the 6 local Commune Health Centers (CHCs). They were recruited through community engagement meetings held by the PHAD team and recruitment flyers posted at the CHCs. Staff at the CHCs, trained by PHAD staff on study objectives, helped facilitate recruitment. Recruitment began in May 2023 and took place over a 1-month period. A total of 642 app users were recruited and invited to download VMood and complete the registration and consent forms embedded within the app. Once app users provided consent, they were asked to self-administer the embedded 9-item Patient Health Questionnaire (PHQ-9), a 9-item depression screening measure, through the app [38]. The PHQ-9 is a widely used short depression screening measure that was selected for this study because it has been validated for use in Vietnam [39-41] and its screening effectiveness compared to traditional (paper-based) formats has been demonstrated on mobile devices [42-45]. Only participants identified with depression caseness as determined by a PHQ-9 score of ≥5 (154/642, 24%) were invited to participate. A PHQ-9 score of ≥5 indicates depression, with scores of 5 to 9 indicating mild depression [38]. We included individuals with mild depression caseness as the VMood app is meant to help individuals with mild to moderate depression. Community women’s union staff assisted app users with app download and registration. Clinical experts familiar with CBT principles were recruited from Thanh Hoa, Vietnam (2/5, 40%), and British Columbia, Canada (3/5, 60%). Experts in Vietnam were recruited through Thanh Hoa’s SPC facilitated by the MOLISA coordinator, with the recruitment process coordinated by PHAD. Experts in Canada were recruited from a local community-based counseling and support services organization identified through similar research projects, along with an expert who was invited based on their familiarity with the in-person SSM intervention. All clinical experts from Canada were native Vietnamese speakers. The recruitment process for all participants in Vietnam was supported by the national MOLISA coordinator based in Hanoi, who has been involved with recruitment and project-related activities throughout the research program (2013-present).

Usability testing involved all participants downloading and testing the app. App users were notified that they had the option of connecting with a commune social worker through a messaging function built into the app for any questions regarding app navigation and basic inquiries about depression. Formative literature on sample sizes for usability testing studies indicates that a sample of 5 participants can identify 80% of a system’s usability issues, additional participants will reveal fewer and less novel usability issues, and the most severe usability issues are likely to have been captured in the first few participants [46].

Exit interviews were conducted with the policy stakeholder and the 2 social workers, and focus groups were conducted with the 13 community women’s union staff members to obtain their feedback on app usability. Focus groups are helpful for elucidating additional information on issues of interest due to interactions and group dynamics among multiple users [47] and can help generate a richer understanding of participants’ experiences and views [47,48], in addition to being time-efficient. Interviews were also conducted with a convenience sample of the 154 app users with depression caseness involved in VMood testing (n=6, 3.9%) based on their availability to gather feedback on their experience using the app. Questions explored the implementation outcomes of acceptability, appropriateness, adoption, and feasibility. Finally, interviews were conducted with the clinical experts to obtain feedback on the conceptual components of the intervention adaptation, along with any strengths and suggestions for improvement.

All interviews and focus groups were conducted in person by the first author (LC), with an interpreter assisting in Vietnam. The interviews lasted between 30 and 60 minutes, and the focus group discussions lasted between 65 and 75 minutes. The interviews and focus groups were recorded, transcribed, and translated into English using forward and backward translation. Detailed field notes during and after the interviews and focus groups were recorded to capture thoughts and observations about the interactions that could help inform the data analysis process [47].

#### App Metrics

Due to technological challenges and the way in which the beta version of the app was designed (reported in the Results section), app use data could not be collected from the participants in the usability testing and, thus, are not reported in this paper. Instead, app use data from the first phase of the RCT, also conducted in the Thanh Hoa province but in a different district, are included. Recruitment in the first 2 communes from the first RCT district in Thanh Hoa began in December 2023 and was completed in March 2024. App use data from these 2 communes are reported in this paper and include data on duration of engagement with the VMood app from 20 app users, all of whom have completed the 3-month VMood program as of June 2024. Data collected using built-in analytics include metrics for evidence on the *adoption* implementation outcome.

#### Discourse Data

The feasibility study also involved an examination of discourse data (meeting minutes, emails, and progress reports) on the app development process that took place from January 2021 to June 2023, along with usability testing in Thanh Hoa from June 2023 to September 2023 and the implementation of VMood in the first phase of the RCT in Thanh Hoa from December 2023 to June 2024. The discussions examined the challenges encountered throughout the various phases of the project and also centered on brainstorming strategies to help ensure continued implementation and success. Certain small-group meetings focused on specific aspects of the app development (eg, technical challenges) or implementation (eg, screening concerns) processes as they arose among select members of the team with relevant expertise.

A main source of data for the app development discussion was the in-person team meetings in Hanoi, Vietnam (August 22-25, 2022). Most members of the geographically distributed research team (from Vietnam, Canada, and Australia) attended. The workshop focused on the app development process, including examining the associated challenges that the team encountered from initial development of the app to subsequent revisions and strategies to help address some of those challenges. All discourse data were either transcribed or captured through detailed meeting minutes and translated where applicable.

Monthly reports on the progress of the first phase of the RCT in Thanh Hoa (monthly reports, n=4) were prepared by PHAD, the implementing partner in Vietnam, detailing implementation updates as well as the challenges encountered and proposed solutions. Challenges included those that the team had experienced in addition to challenges discussed with the provincial implementing partners in Thanh Hoa, who shared their experiences in the community when promoting and implementing VMood. Detailed field notes were also documented throughout the study period by LC to summarize and interpret the various discourse data.

### Data Analysis

Qualitative interview, focus group, and discourse data were analyzed using thematic analysis as outlined by Braun and Clarke [49] to identify and analyze common themes across transcripts that captured important meanings and patterns in the data. A coding framework was developed deductively informed by the outcomes for implementation research framework [50] and also inductively through analysis of the qualitative data. In addition, analysis of focus group data took into account the group dynamics captured via the focus group discussions themselves and researcher field notes. Coding was a multistage process that involved open coding, where preliminary summary statements were provided for elements discussed in the transcript and formed an initial coding framework, and gathering all statements from open coding to collapse into main categories and subsequently into themes, along with validation of the coding by another research team member (senior author JON) in the form of reviewing independently a percentage of the transcripts [51].

App use data were analyzed using basic descriptive statistics.

Data triangulation and reflexivity were used to help ensure the trustworthiness of the data analysis and research findings [52]. Triangulation was used to explore congruence and divergence between data sources, thus increasing the rigor of the findings and reflexivity, aided by the detailed field notes and memos made during the qualitative coding process, to ensure the trustworthiness of the research findings [49]. These strategies provide an audit trail of the analytic process to ensure that other researchers can arrive at a comparable conclusion following the same processes [53]. The audit trail also includes detailed records of the study’s methods and procedures. The NVivo software (version 14; QSR International) [54] was used for all qualitative analysis.

### Ethical Considerations

All procedures were approved by the Research Ethics BC board in Canada (H21-02938) and the institutional review board at PHAD in Vietnam (PHAD-2022/VMOOD-01). Informed consent to take part was obtained from all participants. All VMood app processes for data collection, handling, and storage were encrypted. All interview data were deidentified and stored on a Simon Fraser University–approved cloud storage system. All app data were stored on PHAD’s secure data center using industry-standard 256-bit encryption. The data from this study were coded only using the project name and participant identification number.

The policy stakeholder and providers were given an honorarium equivalent to CAD $10 (approximately VND 185,000 [US $7.36]) for participating in an interview or focus group. App users were provided with CAD $20 (VND 370,000 [US $14.72]) for their testing of the app, along with a phone credit of CAD $3 (VND 56,000 [US $2.21]) for data use on their mobile phones to access the app. Individuals who participated in the interviews were provided with an additional CAD $5 (VND 95,000 [US $3.68]). Finally, clinical experts in Vietnam received an honorarium of CAD $50 (VND 925,000 [US $36.80]) for their participation, including the interview. Clinical experts in Canada received CAD $150 (VND 2,775,000 [US $110.40]). All currency used a conversion rate of CAD $1=US $1.36. The difference in honoraria between experts in Vietnam and Canada was due to differences in the salaries of mental health experts and living costs between the 2 countries and because the ethics committees in Vietnam would not allow for incentives outside their range.

## Results

### Overview

The findings of the feasibility study demonstrated that there were 7 main challenges that fall within the implementation outcomes framework by Proctor et al [34]. Textbox 2 [34] provides a summary.

**Summary of key themes from the feasibility testing results informed by the implementation outcomes framework by Proctor et al.**

**Acceptability**

- Challenges with recruitment and uptake of the app

**Adoption**

- Challenges with use and engagement

**Appropriateness**

- Screening challenges
- Digital divide
- Limitations to digital applications for mental health

**Feasibility**

- Technological challenges
- Funding and policy constraints

### Acceptability: Challenges With Recruitment and Uptake of the App

The first challenge reported was regarding the uptake of the app as social workers and others from the SPC shared that many participants were reluctant to download the VMood app and participate. Reasons cited included resistance to more apps and new technology and security concerns. The policy stakeholder reported that “people are exposed to an abundance of apps; therefore, it is difficult to keep users engaged and accessing the app frequently if there is nothing of interest inside it” and, furthermore, “people in Thanh Hoa ... are reluctant to install unfamiliar apps on their phones out of fear of viruses and malware” (IPS-01), which was another factor impacting participants’ willingness to download and engage with the app. A provider shared the following:

...some people are afraid and worried about their information being leaked.

FGPR-02

App users added the following:

I don’t use others [software/apps] since I don’t find them useful or practical.

IAU-01

Therefore, “the first challenge in terms of getting people to try out this app is to encourage them to download and install a completely new app” [IPS-01].

In addition, the policy stakeholder shared that many individuals interested in the usability testing were not able to participate because their mental health was good according to the PHQ-9 measure and, thus, were ineligible. Many of the individuals were recruited through community events and were quite active in their community and, thus, less likely to have depression. A solution used by the team to address this was the shift to recruiting using a list of high-risk individuals prepared by the communes. A provider suggested the following:

Instead, we could target specifically those who have recently experienced a traumatic event, such as the demise of a family member; then, the likelihood of them experiencing symptoms of depression will be much higher. By doing so, we would be able to reach a group in need of our services and support much more efficiently than if we were to search and look for 1,000 random individuals.

IPR-01

Another provider suggested the following:

...we must consider the commune people’s committee (the administrative centre for government in the commune) and the medical centre, as they are frequently packed with people and have a high likelihood of having someone in need of our services or projects.

IPR-02

Solutions to address these recruitment and uptake challenges centered on using promotional efforts to increase the legitimacy of VMood and awareness of its benefits “to get more people interested in [the] app” (IAU-03). Another app user stated the following:

We must demonstrate the superior features of the app, particularly the application’s features and functionality, so that people understand that using the app is advantageous for them and, as a result, more people are inclined to use it voluntarily.

IAU-04

A social worker reported the following:

...the communication activity to promote this model is quite limited, so sometimes only people in the Project’s region have heard of it, and not many others are aware of it.

IPR-02

An app user mentioned the following:

...in order for this software to reach a wider audience, communication and propaganda processes for people must be emphasized...When they understand how to use the app and enjoy using it, they will tell others, and ultimately, everyone will start using it.

IAU-06

Strategies included sharing more information and education through Zalo (Vietnam’s messaging platform) and Facebook group chats of the commune’s various unions (eg, women’s, youth, and pregnant women unions) and by combining with promotional strategies for other initiatives. The team has also created a series of promotional broadcast messages to be sent via Zalo and Facebook groups to help address concerns among the community about scams and phishing.

Participants also spoke about the importance of increasing the visibility of government support to encourage trust and uptake, especially in the context of increased scams orchestrated through digital media such as smartphones. For example, a provider indicated the following:

I think it [VMood] should have a QR code or an official, legal advertisement for this app; and the site where we place promotion signs or posters, or where we help users in installing this software, should be a government-related and trustworthy location, such as a medical station. [Otherwise,] people might think it’s a scam.

FGPR-01

An app user echoed this, stating the following:

...people today frequently have faith in initiatives that receive support from the Authority. So if it is protected by the government, people will certainly follow and try it out.

IAU-04

There was a recommendation from the provincial SPC for MOLISA to dispatch notifications to the provinces to clarify the information on VMood and increase trust in the app. Another provider shared their perspectives from a different angle:

As a professional, I have no right to impose social issues (such as forcing them to install the app) on people. However, if we promote the use and installation of this app through and with the assistance of the government, local authorities, and the district or commune-level government, then participation will undoubtedly increase.

FGPR-02

Another challenge to recruitment was regarding low mental health awareness and stigma. SPC staff reported that commune health staff at times introduced the project using words that held sensitivity in the community, such as mental illness, which people still associate with schizophrenia. Consequently, some individuals in the community were afraid of being discriminated against and, therefore, were reluctant to participate in the project. To help address this, the PHAD team has prepared an introduction sheet for the local commune health staff to refer to when recruiting participants.

Promisingly, participants in all 3 groups from the usability testing spoke about the convenience and general acceptability of VMood in their communities, indicating that it was simple, easy to use, and suitable for all ages. Once individuals install the app, “from then, people will then begin using and recognizing the app’s utility and usefulness” (IPS-01).

### Adoption: Challenges With Use and Engagement

Findings from the qualitative interviews and focus groups indicated that app user engagement was fairly low. The first factor influencing participant’s adoption of VMood was the busy schedules of the app users. While they reported enjoying the app and certain features, including the “multiple-choice questions” and videos, one app user indicated the following:

I haven’t watched all of the videos because I do not have sufficient time to watch them all.

FGPR-02

Another participant similarly reported the following:

I was able to use the app perhaps 2-3 times [in a month]. It’s not that often because I don’t have a lot of time due to my workload.

A provider mentioned that people working in agriculture may not find the app as acceptable as perhaps a younger participant because of their busy schedule during “agricultural seasons,” where “people will be preoccupied with work or study,” which may result in “an overall drop in their interest in mental health” (IPR-01).

Strategies to increase participant engagement included incentives such as “monetary support” (FGPR-01) in the form of a phone card or, as another provider from the same focus group mentioned, “a form of award that could be turned into cash every time a user correctly answers a question.” This provider recognized that “the project won’t be able to offer monetary assistance endlessly” but perhaps “could opt to keep it simple, such as 10 points=VND 10,000 (CAD $0.50).” They concluded that “this would very certainly encourage users to open the app and use it more frequently.” Another strategy was suggested by PHAD, informing participants that they would be entered into a draw for an Android smartphone.

Another strategy suggested was “a management team, or group leader” who “should be assigned to each locality. They could be in charge of contacting or reminding people [from that area] through this app” (FGPR-01). The participant clarified that “an administrator account must be created for the medical station so that they can administrate their patients” and “keep track of them.” Another provider from the first focus group spoke about this need, indicating that, in their commune, there was “a large number of people who have installed this app; nevertheless, the management provided to manage and follow up with this group is insufficient” (FGPR-01).

App use data from the early phase of the Thanh Hoa RCT implementation showed that more than half (12/20, 60%) of the app user participants had high levels of engagement with the app (>120 minutes), 30% (6/20) had moderate levels of engagement, and only 10% (2/10) of the participants engaged with the app for <60 minutes since enrollment. It should be noted that use for app users was tracked beyond the 3-month intervention period for an additional 3-month follow-up period (total of 6 months). These data, which were last extracted on June 24, 2024, showed that, since enrollment, the 20 app user participants engaged with the app, on average, for 145.9 (SD 96.2) minutes, with the highest engagement being 366 minutes and the lowest being 13 minutes (median 125.5, IQR 83.5-168.25, range 13-353). These findings suggest that some of the strategies to increase participant engagement may have been effective [55]. For the purpose of this study, the engagement cutoffs were established based on preliminary interviews exploring engagement with app users in the RCT from 2 provinces, and the clinically significant treatment dose will be investigated further in the RCT.

### Appropriateness

#### Screening Challenges

The first challenge reported that relates to VMood’s appropriateness was regarding screening. Thanh Hoa SPC participants reported that some individuals responded falsely to the embedded PHQ-9 screening measure to gain access to the app. Certain app users who were invited to the usability testing initially scored low on the PHQ-9 and, per the study protocol, were asked to retake the PHQ-9 in 2 weeks. Some participants then asked for and received instructions from the commune health staff on how to obtain access to the app by scoring high on the PHQ-9. Thus, many participants in the usability testing with depression as measured by a PHQ-9 score of ≥5 (n=154) did not, in fact, have depression. Commune health staff confirmed that the 3.9% (6/154) of app users who participated in the interviews were true positives. This screening challenge was discussed at team meetings, and it was decided that the full version of the app would be provided to all app users regardless of depression caseness, so there would be no need for faking their PHQ-9 scores. Another suggestion was made to communicate clearly to the communes on the intervention benefits for those who need it.

#### Digital Divide

Another challenge that impacted VMood’s appropriateness was the digital divide. Although most participants commented on the strengths of the VMood digital intervention in reaching a broader population, there were concerns raised regarding the digital divide—namely, how certain populations may be left behind. For example, a clinical expert shared the following:

I can see the app can be very applicable to my generation who are quite familiar with using apps and internet to navigate life situations. But for my parents’ generation, for example, using this app can prompt a lot of anxiety for them to be logged in and where are the buttons and all that.

IEX-05

Similarly, an app user stated the following:

...senior citizens ... have a more difficult time processing than young people like me.

IAU-03

Another app user also commented on their parents, who “are not proficient with technology” and may not be able to log in and “use this app on their own.” This was similar to the in-person RCT, where older individuals had difficulty reading the ASW and asked their family members to help read to them. Despite this, this app user indicated that they would introduce the VMood app “if any family members needed it.”

Other participants also commented on structural barriers to widespread implementation and uptake of VMood. One provider reported the following:

As urbanization expands to the countryside, a group of young people might be affected from the use of the Internet.

FGPR-01

Another provider reported the same concern regarding internet connectivity issues:

In some locations, the connection or Wi-Fi signals are inadequate or unstable, which could have an effect on the implementation of this project.

IPR-01

Despite potential issues with Wi-Fi signals, it was reported by the Thanh Hoa leadership that Wi-Fi is available in most homes because television typically runs on internet connection, delivered through Wi-Fi. Similarly, 4G is available in most households except for some individuals from older generations. In addition, the project provides app users with a phone credit for their participation.

The team discovered that a larger number of individuals than anticipated did not own newer smartphones. An app user mentioned the following:

...while technology coverage in Vietnam is high, the proportion of the population owning smart devices is limited.

IAU-06

As a result, they “rarely have the opportunity to interact with (VMood)” (IEX-05). A clinical expert shared that “farmers, for example, do not know how to use smartphones and do not even own them ... if you move further away from the city, then you would see that not everyone knows how to use them.” Another example of lack of smartphone ownership was given by a provider, who explained that “it can be difficult to install the app if a family has 2-3 members who are depressed, but only one of them has a smartphone” (FGPR-02). The decision made after discussing this with the SPC staff and staff at the local communes was to recruit only those with smartphones for the RCT as it would not be feasible to provide smartphones to all app user participants.

Finally, the team also learned during the usability testing that VMood did not always function properly on older smartphones that were not able to support newer apps. After discussing with the app development company, they confirmed that this limitation was due to Apple and Android providers that do not support newer software, which means that VMood will likely be available only to users of newer smartphones.

#### Limitations to Digital Applications for Mental Health

##### Overview

Another challenge influencing VMood’s appropriateness was limitations to digital applications for mental health. These included the complexity of the app, such as the CBT principles VMood is based on, along with cultural and linguistic challenges. The first point participants spoke about was the complexity of the app. While one provider from the first focus group indicated that “in my opinion, the installation process is relatively easy and convenient for most people” (FGPR-01), another provider from that focus group, who indicated that they were a “collaborator [community women’s union staff] who provides advice to people ... making it easier for them to access the app,” stated the following:

The initial installation phase is easy and convenient for people to follow, but because this app is quite new, the app has not yet reached a high level of usage. Therefore, I believe that this initiative still requires collaborators to assist users in the process of using this app to evaluate their health.

FGPR-01

App users similarly mentioned the following:

It might be difficult if I let myself figure it out because it isn’t written on the software about what to do or why. How can I accomplish it if I don’t know what it is?

IAU-05

App users also spoke about the complexity of the VMood content. For example, one app user indicated that, while they enjoyed watching the videos on the app, “the explanation for the app’s content is a concern. I’m not sure if it’s because of my age or my academic level, but there are several things in the app that I have trouble understanding” (IAU-02). Suggestions were provided by participants to help reduce some of these concerns. A clinical expert proposed the creation of “a group of the people who are using the app and having a little talking circle ... empowering each other” (IEX-04).

##### Cultural and Linguistic Challenges and Suggestions

Despite the ASW being tailored to fit the local context, participants shared feedback on including more culturally appropriate examples for app components, such as components on reactivating one’s life, a food guide, and creating a better sleep environment (eg, “including traditional local remedies for sleep” [IEX-03]), to improve VMood’s cultural appropriateness. One clinical expert mentioned that the app is focused more on the “brain,” but “Vietnamese people, they can be superstitious” (IEX-04). Another participant remarked the following:

...a few questions only apply to a specific group of workers such as office workers, whereas lots of Vietnamese are freelance or manual labor. ... The context of the question should be relevant and close to a normal daily life of a Vietnamese.

FGPR-01

Another provider from the same focus group suggested the following:

...the next training session at the centre should be tailored to suit the local area.

FGPR-01

This was noted as being especially important considering that many of the participating districts in Thanh Hoa province are “mostly in rural areas.” Finally, a provider spoke about the implementation and scale-up challenges posed by cultural and linguistic issues:

I imagine that each region will face distinct difficulties, such as mountainous regions and remote islands. Because we are discussing a nationwide implementation of this app, each region will face particular difficulties. For instance, it might be challenging to introduce a Kinh-language app in areas where people speak ethnic languages. These are the difficulties I could imagine if we were to deploy this application nationwide.

IPR-01

The team reviewed the suggestions, and Vietnamese-speaking members of the team, consulting with a social worker living in a rural community, added more culturally relevant examples for the realistic thinking and problem-solving skills components to increase appropriateness. Promoting VMood acceptability and usability requires an understanding of and tailoring to local traditions and culture, which participants indicated will be a key factor to its sustainability across Vietnam’s diverse communities, each with its own unique needs.

### Feasibility

#### Technological Challenges

Technological challenges included delays experienced with the app development process and missing app use data from the usability testing. The team wanted to ensure that VMood had theoretical fidelity to the original in-person intervention and practical fidelity in terms of usability in its digital format. The study by Chau et al [35] provides the results of fidelity testing. As a result of the technological challenges, the development process was long and required frequent exchanges among the larger project team, our implementing partners in Vietnam who were working directly with the app development company, and the app development company. Any edits made to the app also required testing and fixing any bugs in addition to approval from the Apple App Store. This further delayed the editing process, sometimes causing updates to take >2 months to go live. Developing the app was a major undertaking that required significant input and resources.

Some participants from the usability testing indicated that the text size was a bit small, especially for older adults, and suggested increasing the font size. The team discussed this with the app development company and agreed to delay this change until after the RCT is launched in Thanh Hoa as making this update would require weeks and, furthermore, with an increase in font size, the text may flow out of the frame on smartphones. Another issue experienced during the usability testing was that the instructional videos lagged in some areas with slower internet speed. To help mitigate this, YouTube was chosen as the video hosting platform as it can adjust the video stream quality based on the user’s internet connection.

Another technological challenge encountered was with the technical complications and oversight in the original app design despite having a sophisticated design process in place. The app did not have the functionality for capturing app use data, including the number of log-ins. We were under some pressure from the Government of Vietnam to launch VMood fairly quickly to meet their fiscal timelines, and the app design company had not yet set up the app to capture use frequency and duration. We were made aware of this after usability testing had been completed and, thus, were not able to include any app use data from the usability testing participants. The app was subsequently updated to include built-in metrics to capture use data such as frequency and duration of engagement with the various skill components.

#### Funding and Policy Constraints

Funding and policy constraints were the last challenge that the team encountered. Social workers participating in the larger RCT will provide support as part of their regular job responsibilities, which typically involve a heavy workload. Thus, their time will be limited for both training and project implementation. Some social workers commented on the limitations to the training program:

Regarding in-depth training or how to deliver counseling for a case of depression, we have not received such training. We have not received specialized training on depression; we have only received training related to the book, including how to use the book/handouts and how to assist others in using/reading this book. ... I am interested in attending other in-depth training sessions on this topic if they are offered.

IPR-02

Despite this, social workers indicated that they were satisfied overall and appreciative of the training and mentioned that, before receiving the training provided by project team members on how to administer the intervention and on basic depression, they had received little or no training on depression. For example, one provider stated the following:

I thought the training session was very sociable and enjoyable, and I learned some new things [about depression] that I had never known before.

FGPR-01

Another provider also reported that the training program was “quite handy. I was able to gain extra knowledge and also use what I learned in these sessions for my everyday tasks” (IPS-01). Furthermore, for VMood installation and navigation, which are the social workers’ main functions on this project, a provider shared the following:

If we are only discussing whether the training is sufficient to allow us to assist users with accessing and using the application, then I believe the training materials are adequate.

IPS-01

Other social workers also shared that they had limited capacity to provide extensive support through the app. The app is not meant to be a magic bullet for everything but is intended as a widely accessible tool for those with mild to moderate depression, with minimal support on app navigation and basic depression provided by social workers.

High turnover of social workers posed a challenge for the feasibility testing, slowing down implementation in the early phase of the RCT in Thanh Hoa as there was a need to train new social workers fairly frequently, but the team has developed clear training guidelines supporting the web-based training program, and provincial project managers will be responsible for training new personnel carefully according to the guidelines. In addition, the director of Thanh Hoa’s SPC changed just before the expected launch of the RCT, which also contributed to a delayed RCT launch in Thanh Hoa.

Despite the challenges described previously, ultimately, most participants indicated that the VMood app is an important tool that can assist individuals in the community experiencing depression. Strengths reported for the digital intervention included being able to use the app privately “at home” (IEX-01) in their own environment, potentially mitigating some of the effects of stigma. One clinical expert stated the following:

It’s great because depressed patients frequently don’t want to tell anyone about their problems, but they can download this app, watch it, understand it for themselves, and change their mindset.

IEX-02

In addition to the ability to use VMood in the comfort of participants’ environment and at their convenience, another advantage is the ability of VMood to “reach a broader audience” (FGPR-01).

A provider who had been involved with the RCT for the in-person intervention compared it with the digital version and shared the following about the previous intervention:

Since the release of the VMOOD software, I’ve realized that it’s much better in terms of security. Because previously we had to send social workers to people’s homes to ask questions, we no longer need to do so, and users can now experience this software on their own. This will reduce the likelihood of people being shy while answering the PHQ-9 questionnaire, which could contribute to inaccuracy. ... In the past, when we went to collect people’s responses, they sometimes responded differently despite their obvious illness behavior.

IPR-02

In summary, participants expressed unanimous support for the VMood intervention and highlighted the need for increased community-based mental health care because, currently, “there is no infrastructure in Thanh Hoa to support these people” (IPR-01). This same provider reported that “because in today’s developed, modern society, life and work are fraught with pressures and difficulties. Consequently, I believe that numerous people are struggling with their mental health. I believe that many people will be interested in this topic.” A clinical expert also recognized the limitations to the current mental health care system and shared that they hoped that the intervention would be scaled up to assist more people to “relieve some of the burden on [them]” (IEX-01). The Government of Vietnam recognizes the gaps in community-based mental health care and has provided ongoing support, including commitment in the form of matching funds, to support VMood implementation and scale-up.

## Discussion

### Principal Findings

Feasibility testing of the VMood app demonstrated a number of key challenges to its development and implementation that fell within 4 of the implementation outcomes by Proctor et al [34]: acceptability, adoption, appropriateness, and feasibility. Recruitment challenges arose that impacted VMood’s *acceptability*; app use and engagement challenges impacted its *adoption* in the community; screening challenges, along with considerations regarding a digital divide and limitations to digital applications for mental health, impacted VMood’s *appropriateness*; and, finally, technological challenges and funding and policy constraints needed to be considered to ensure VMood’s *feasibility* within the Vietnamese context. Extensive discussions at various meetings throughout the app development process, usability testing, and launch of the RCT in Thanh Hoa among team members and with leadership at the Thanh Hoa SPC helped the team fully understand the challenges and brainstorm timely solutions to help mitigate them.

### Comparison With Prior Work

Participants found VMood to be acceptable within the Vietnamese context, although there were extensive challenges reported with recruitment, such as resistance to more apps and new technology, security concerns, and persisting low mental health awareness and stigma. Similarly, other studies have shown that data privacy and security are concerns that impact acceptability [56]. Mental illness stigma is high in Vietnam, as in many other Asian countries [57], but promisingly, depression app use has been shown to increase mental health literacy and awareness and mitigate the effects of stigma as people are able to access them in their own environment and experience increased anonymity [58]. A main solution to enhance the acceptability and legitimacy of VMood was to increase promotional efforts, including sharing more information and education through social media and exhibiting government support more explicitly on promotional materials. Usability testing also highlighted the importance of a targeted recruitment approach using lists prepared by commune health staff of individuals considered to be at a higher risk of developing depression based on recent adverse life changes. This strategy appears to have been effective in the first phase of the RCT. Participants also reported on some challenges to adoption, including the busy schedule of app users, which may prevent them from fully engaging with the app, especially during busy periods such as harvesting season. Incentives in the form of money and a chance to win an Android smartphone were incorporated for the RCT to encourage participant engagement. Finally, the specific symptoms of depression, such as low motivation, concentration difficulties, and behavioral avoidance, may pose additional challenges to app engagement [22]. For example, Arean et al [55] found that participants with a higher baseline depression score (measured using the PHQ-9) accessed their 2 depression apps less frequently, suggesting that depression apps may have the greatest impact on individuals with milder levels of depression [55]. Screening through depression apps could help provide an indicator of those who require and should be prioritized for more in-person treatment. App use metrics from the early phase of the Thanh Hoa RCT indicate that 60% (12/20) of the participants had high engagement with VMood (>120 minutes), 30% (6/20) had moderate engagement (60-120 minutes), and 10% (2/20) had low engagement (<60 minutes). A closer comparison between app user engagement and their PHQ-9 scores is warranted and will be conducted in the larger RCT.

The appropriateness of VMood was impacted by screening challenges in which a number of participants responded falsely to questions in the embedded PHQ-9 to gain access to VMood. Data integrity has been reported as an increasing concern in web-based research [59], defined as “any research involving the remote acquisition of data from or about human participants using the internet and its associated technologies” [60]. Specific concerns relevant to this study include nongenuine participants, which is when participants lie about their experiences or identity, and misrepresentation, when participants exaggerate specific details [59]. Possible motivations for participants falsely responding to the PHQ-9 include gaining access to the intervention and monetary incentive [59]. As discussed in the Results section, one strategy that the team decided to use to help address this was to provide the full app to all app users who signed up. There are also considerations that need to be taken for certain groups (eg, older adults) to ensure equitable access for all who might benefit and not further increase the digital divide, which is “a division between people who have access to and use of digital media and those who do not” [61]. The last challenge impacting appropriateness was limitations to digital applications for mental health. Lower-income countries have experienced faster mobile phone use growth rates that higher-income countries [58]. For example, Vietnam, a country of approximately 100 million people, has approximately 140 million mobile telephone subscriptions [62]. Despite this, it was found that many individuals in the communities did not, in fact, own smartphones or owned older versions that were not able to support newer apps. In addition, for a tool that is meant to be widely accessible and available, there were issues reported on the complexity of the app, including the CBT principles and cultural and linguistic concerns. These were addressed by encouraging participants to review the instructional videos thoroughly and incorporating more culturally relevant examples, respectively. The structured approach of CBT translates well to a mobile format [63], and with the accompanying videos and any necessary support provided by a social worker, community leaders were confident on VMood’s appropriateness. Cultural appropriateness is a key consideration when adapting an intervention from one context to another. The study by Chau et al [64] provides an in-depth discussion on cultural fidelity. There is a growing understanding of and attention to ensuring culturally appropriate mental health care for people in diverse settings [65]. Despite some of the challenges reported on the complexity of the app, participants did not comment on some of the more common hurdles to usability found in the literature, such as the tool requiring too much time to enter data or being irrelevant to their needs [66].

As discussed, a main challenge encountered in this study was related to technological issues resulting in missing app use data from the usability testing. Despite this, through interviews and focus groups, usability testing participants were unanimous in expressing their support for the app and provided positive feedback on various components of the app, such as the instructional videos and realistic thinking skills. Interviews and focus groups are 2 of the most commonly used methods for usability testing, in addition to questionnaires and use data, and allow for the elucidation of users’ experiences and the likeability of the app in addition to barriers to use and ideas for improvement [67]. App users also indicated that they would recommend VMood to friends and family based on their experiences up to the date of their interviews. App metrics from the first phase of the RCT launch in Thanh Hoa complemented the qualitative reports from the usability testing participants and showed that 60% (12/20) of the app users demonstrated high engagement with the app (>120 minutes), suggesting that they found it to be helpful for their needs.

In relation to the feasibility of VMood implementation, participants also commented on the importance of having support from social workers through the app and corresponding social worker training to ensure that they are adequately prepared to provide basic support. A systematic review examining the effects of mobile apps on stress, anxiety, and depression similarly found that integrating human support into digital health interventions has been shown to increase usability [24] and produce larger clinical effects than those of stand-alone apps [68]. Participants spoke about the importance of social worker support but also recognized the need for a training program that can facilitate rapid deployment of social workers and agreed that a balance needs to be struck between feasibility and appropriateness within current budgetary and policy constraints. It is important to recognize the concurrent responsibilities of social workers, who were participating in this project as part of their regular work duties, and avoid overburdening them, which in turn can contribute to high stress and high turnover.

Presently, evidence on the effectiveness and cost-effectiveness of smartphone-based apps is fairly limited [21,69], with at most only approximately 4% being evidence based [70]. There is also limited evidence on whether effectiveness studies are impacted by failed implementation [71]. Implementation science focuses on practical approaches to enhance the scale-up and sustainability of various evidence-based interventions [72] by helping bridge the longstanding knowledge-practice or know-do gap [73]. The various challenges reported during VMood development and implementation, along with solutions to address them, will be critical as the research team conducts the RCT across the remaining 7 provinces (this work is ongoing) to help avoid similar challenges. Suggestions from the usability testing participants, leadership in the communities, and the research team may help mitigate the high dropout rates and low engagement with technology that are increasingly recognized as challenges for digital health interventions in terms of both RCT validity and sustained engagement [66]. The findings of this feasibility study will also contribute important evidence informing other research examining the development and implementation of similar digital interventions for mental health. However, it is important to recognize that certain strategies to increase recruitment and engagement, such as providing app users with a phone credit for their participation and entering them into a draw for an Android smartphone, were necessary and suitable for the RCT but may not be feasible for scale-up. Thus, should the RCT demonstrate VMood’s effectiveness, other strategies supporting real-world implementation beyond the context of an RCT will be required for its scale-up. Ultimately, despite these challenges and limitations, participants from the usability testing spoke unanimously about the importance of a low-cost and accessible intervention such as VMood that could help fill a growing treatment gap in the community, where mental health conditions are prominent and on the rise, especially in the context of the COVID-19 pandemic [3].

### Limitations

Due to challenges encountered during the development, feasibility, and usability exploration of the VMood app, we were unable to carry out the feasibility study as originally planned. However, this formative paper focuses on the immediate strategies and adjustments that the team used to address the challenges, taking advantage of a unique opportunity to share the lessons learned regarding the development and feasibility testing of a digital mental health intervention that we believe could be helpful for others similarly interested in developing and testing similar interventions. In addition, while the team used strategies to help ensure cultural and linguistic appropriateness capturing Vietnam’s diversity, such as conducting beta testing with individuals familiar with different dialects, we recognize that it may be difficult for 1 app to fully capture Vietnam’s diversity. Should RCT findings demonstrate VMood’s effectiveness, we will adapt the app to fit different Vietnamese dialects and subgroups (eg, ethnic minority groups and individuals living in the industrial zones) and subsequently examine data across the different subgroups.

### Conclusions

Adjustments based on recommendations critical to VMood’s usability were made to the app, which is currently being tested in an RCT across 8 provinces of Vietnam. The RCT will examine VMood’s effectiveness along with cost-effectiveness. The work by Chau et al [74] provides the RCT protocol. The Government of Vietnam has provided ongoing support for this program of research as part of a strong and sustained research-policy collaboration since 2013 (see the work by Murphy et al [75] for further details) and has committed to scale-up in the other Vietnamese provinces should results demonstrate effectiveness. The findings of this implementation science feasibility study contribute important evidence on the challenges to the development, feasibility, and usability testing of a digital depression app adapted from an in-person intervention in an RCT in Vietnam, a low- to middle-income country. The findings have applicability for others looking to develop and implement digital interventions in similar contexts, particularly given the demand for digital mental health solutions to expand mental health care, serving as a unique opportunity to share the lessons that our team learned regarding the development and testing process.

## Abbreviations

ASW: Antidepressant Skills Workbook
CBT: cognitive behavioral therapy
CHC: Commune Health Center
MOLISA: Ministry of Labour, Invalids, and Social Affairs
PHAD: Institute of Population, Health, and Development
PHQ-9: 9-item Patient Health Questionnaire
RCT: randomized controlled trial
SPC: Social Protection Center
SSM: supported self-management

## Data Availability

The datasets generated or analyzed during this study are available from the corresponding author on reasonable request.
